# Drug–Drug Interactions of FXI Inhibitors: Clinical Relevance

**DOI:** 10.3390/hematolrep16010016

**Published:** 2024-03-21

**Authors:** Nicola Ferri, Elisa Colombo, Alberto Corsini

**Affiliations:** 1 Department of Medicine, University of Padova, 35128 Padova, Italy; 2 Veneto Institute of Molecular Medicine (VIMM), 35129 Padova, Italy; 3 Coordinating Research Centre on Pharmacological Interactions, University of Milan, 20133 Milan, Italy; elisa.colombo1@unimi.it (E.C.); alberto.corsini@unimi.it (A.C.); 4 Department of Pharmacological and Biomolecular Sciences “Rodolfo Paoletti”, University of Milan, 20133 Milan, Italy

**Keywords:** DOAC, FXIa, mAbs, ASO, drug–drug interactions

## Abstract

Inhibitors of the factor FXI represent a new class of anticoagulant agents that are facing clinical approval for the treatment of acute coronary syndrome (ACS), venous thromboembolism (VTE), and stroke prevention of atrial fibrillation (AF). These new inhibitors include chemical small molecules (asundexian and milvexian), monoclonal antibodies (abelacimab, osocimab, and xisomab), and antisense oligonucleotides (IONIS-FXIRX and fesomersen), and thus, they have very peculiar and different pharmacokinetic and pharmacodynamic properties. Besides their clinical efficacy and safety, based on their pharmacological heterogeneity, the use of these drugs in patients with comorbidities may undergo drug–drug interactions (DDIs) with other concomitant therapies. Although only little clinical evidence is available, it is possible to predict clinically relevant DDI by taking into consideration their pharmacokinetic properties, such as the CYP450-dependent metabolism, the interaction with drug transporters, and/or the route of elimination. These characteristics may be useful to differentiate their use with the direct oral anticoagulant (DOAC) anti -FXa (rivaroxaban, apixaban, edoxaban) and thrombin (dabigatran), whose pharmacokinetics are strongly dependent from P-gp inhibitors/inducers. In the present review, we summarize the current clinical evidence on DDIs of new anti FXI with CYP450/P-gp inhibitors and inducers and indicate potential differences with DOAC anti FXa.

## 1. Introduction

Direct oral anticoagulants (DOACs) have represented an innovative strategy for anticoagulation therapy as an alternative to warfarin and heparins. The therapeutic class of DOACs include one thrombin (Factor II activated, FIIa) inhibitor, dabigatran, and three FX inhibitors, rivaroxaban, apixaban, and edoxaban. DOACs are indicated for prevention of stroke in non-valvular atrial fibrillation (NVAF) patients, venous thromboembolism (VTE), and for treatment of acute coronary syndrome (ACS). Since the first approval in 2010, DOACs have shown in randomized, placebo controlled, clinical trials the same efficacy as warfarin in preventing thrombotic events but with approximately 50% lower risk of intracranial bleeding events [1]. This advantageous safety profile is partially limited by a 25% increased risk of gastrointestinal bleeding compared to warfarin [1]. The administration of DOACs is also more convenient than heparins and warfarin for use at fixed doses without routine coagulation monitoring. Despite their improved safety profile and compliance, DOACs still have limitations, including the high risk for gastrointestinal bleeding and relevant drug–drug interactions (DDIs) with other therapies [2].

In search of new anticoagulant agents with a better clinical profile, the inhibition of FXI has been considered very attractive in consideration of the reduced risk of thrombosis with minor bleeding tendency of subjects carrying a genetic defect of this coagulation factor [3,4].

**Figure 1:**
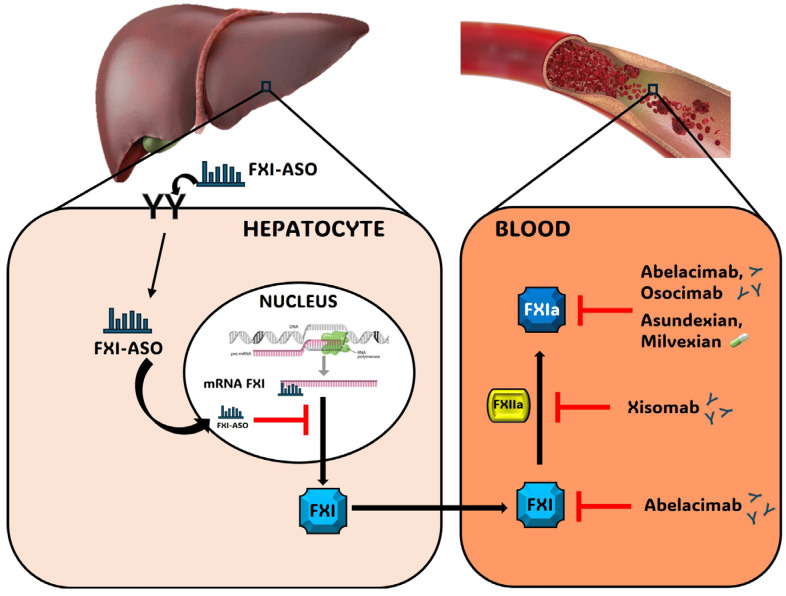
Schematic representation of the molecular mechanism of action of the anti FXIa under development. Chemical small molecules (asundexian and milvexian) and monoclonal antibodies (abelacimab, osocimab, and xisomab) inhibit circulating FXIa and/or its activation, while antisense oligonucleotides (IONIS-FXIRX and fesomersen) block its transcription in the hepatocytes.

In the new era of pharmacology, it is imperative to use different strategies for a proper inhibition of a particular molecular target, i.e., FXI. Currently, there are three classes of drugs directed against FXI under clinical development: small molecules (asundexian and milvexian), monoclonal antibodies (abelacimab, osocimab, xisomab), and antisense oligonucleotides (ASOs) IONIS-FXIR_X_, and IONIS-FXI-LR_X_ (Figure 1).

Promising results of phase 2 trials in patients undergoing major orthopedic surgery and in those with end-stage renal disease (ESRD), NVAF, and ACS have led to large phase 3 trials that are currently ongoing [5,6]. In completed phase 2 clinical trials, FXIa inhibitors were compared with current anticoagulant strategies (i.e., low molecular weight heparin or apixaban). The results suggest a favorable safety and efficacy profile for FXI/XIa inhibitors but generally fail to demonstrate a significant reduction in thrombotic occurrence, probably due to the small, randomized trials that were not powered for the efficacy endpoint [7,8,9,10,11,12]. The benefit/risk ratio of these new drugs remains to be confirmed in phase III large studies. Among these, the OCEANIC-AF phase III study has been stopped early due to inferior efficacy of asundexian versus apixaban for preventing stroke in patients with NVAF. Since the available safety data are consistent with previously reported safety profiles of asundexian, the drug is still under investigation in the OCEANIC-STROKE phase III study as planned [13].

The fully human monoclonal antibody (mAb) abelacimab binds and inhibits both FXI and FXIa [14], while osocimab specifically blocks FXI in the active configuration (FXIa) [15]. Differently, xisomab inhibits the activation of FXI by FXIIa rather than inhibiting FXIa activity or activation of FXI by thrombin (Figure 1). These drugs are administered by i.v. or s.c. injection and have a longer elimination half-life compared to small oral drugs (25–40 days) [15].

Asundexian and milvexian are chemically synthesized molecules, which potently inhibit the active form of FXI (FXIa) in a reversible manner. Asundexian show a relatively longer half-life than milvexian (15 h vs. 10 h). Accordingly, asundexian is administered orally once a day while milvexian can be used also with twice daily posology [15].

Different from small molecule drugs or biologics, which bind and affect target proteins, ASOs are short chemically modified oligonucleotides that bind to complementary RNA in cells and modulate the RNA function to produce a pharmacological effect [16] (Figure 1). IONIS-FXIRX (BAY2306001) is an ASO that inhibits the synthesis of coagulation factor XI (FXI), while FXI-LICA (BAY2976217, IONIS-FXI-LRX, fesomersen) shares the same RNA sequence as IONIS-FXIRX but contains a GalNAc-conjugation that facilitates asialoglycoprotein receptor (ASGPR)-mediated uptake into hepatocytes. This approach is useful since the liver is the primary source of plasma FXI [17]. The ASOs are administered subcutaneously and show a half-life of elimination of approximately 1–2 weeks [18]. The GalNAc-conjugation, present in the FXI-LICA, has a strong influence on the pharmacokinetics and pharmacodynamics, allowing lower and less frequent dosing (once-monthly vs. once-weekly) to yield comparable pharmacological activity as unconjugated ASOs [19] (Table 1).

## 2. Pharmacological Considerations of Drug–Drug Interaction with mAbs and ASOs

To predict the possible drug interactions of mAbs and ASOs with small chemical drugs, it is mandatory to know their metabolic pathways and elimination processes. On the other hands, the absorption phase can be excluded from the prediction of possible drug interactions since both types of drugs are administered subcutaneously.

Abelacimab, osocimab, and xisomab, like other mAbs, are eliminated by means of catabolic processes. mAbs are catabolized into peptides and amino acids primarily by the reticulum endothelial system (RES), which consists of phagocytic cells, such as macrophages and monocytes [20]. For this reason, their elimination is mainly independent from the liver and kidney function. A second relevant route of elimination is target-mediated, consisting in the internalization into cells expressing the receptor and/or cell surface protein that is recognized by the antibody, which follows intracellular catabolism within the lysosomes. However, this mechanism of elimination can be excluded for mAbs targeting freely circulating factors, such as the anti FXIa. For the same reason, the elimination of mAbs anti FXIa can be predicted to be constant, independently from the dose. Indeed, target-expressed cells are limited in number, and their capacity to eliminate mAbs is saturable and, therefore, decreases with the dose of antibody administered. On the other hand, antibodies directed to freely circulating factors cannot undergo target-mediated elimination, but mainly through the RES, and thus, their clearance is constant and independent by the dose. However, some exceptions have been described, with mAbs that, even if they are targeted by freely circulating antigens, may undergo dose-dependent clearance. The freely circulating mAb-antigen complex can be recognized by the Fcγ receptors expressed on monocytes and macrophages, which subsequently triggers its internalization and catabolism [21,22].

Finally, it must be considered that the administration of therapeutic mAbs can lead to the generation of autoantibodies by the immune system. The antibody response still represents a clinically relevant event that may interfere with the pharmacological activity of mAbs and contribute to their clearance [23,24]. The activation of the antibody response depends on numerous factors including the state of the patient’s immune system, the co- administration of other drugs, the structure and the antibody composition (glycosylation), but, more importantly, on the similarity with the endogenous IgG [25]. Usually, fully human antibodies are less immunogenic than humanized ones, a difference that had determined, for instance, the failure of the clinical development of the anti PCSK9 antibody bococizumab [26].

After a single dose i.v. and multiple dose s.c. of abelacimab, no anti-drug antibodies (ADA) have been detected, confirming the non-immunogenicity of this fully human mAb [27]. Conversely, after single i.v. or s.c. doses of fully human mAb osocimab, ADA formation was confirmed in 6 volunteers out of 56 included in the study [28]. In phase 1 study, xisomab, a humanized anti-FXI antibody, did not show immunogenic reactivity with subjects with anti-drug antibodies detected [29]. The apparent higher immunogenicity of osocimab, compared to xisomab, may have a potential impact on its clearance that can be reduced in patients treated with immunosuppressive drugs, such as methotrexate, azathioprine, or mercaptopurine, as observed with infliximab and adalimumab [30,31].

ASOs are synthetic single-stranded nucleic acids designed to complementary hybridize to target transcripts and induce their degradation by means of RNase H1 [19]. Since ASOs typically show broad distribution into different organs, their conjugation with triantennary *N*-acetyl galactosamine (GalNAc) delivers specifically the antisense to hepatocytes through high-affinity binding to asialoglycoprotein receptor (ASGPR). The absorption of ASO and GalNAc-conjugated ASOs is rapid following subcutaneous injection with a time to maximum concentration (T_max_) between 1 and 4 h [19]. GalNAc-conjugated ASOs are highly bound to plasma proteins, and the extent of protein binding is mostly greater than 98%. The GalNAc conjugation showed good stability in blood after absorption; although, metabolites with 1, 2, and/or 3 GalNAc sugar deletions or unconjugated ASOs can be detected. The total exposure of each of its metabolites accounted for less than 13% of the total full-length oligonucleotide [32]. The area under plasma concentration–time curve (AUC) for unconjugated ASO accounted for ≤5% of the total full-length oligonucleotides within the first 24 h post dose. After 24 h, the unconjugated ASO became the major circulating species in the plasma [19]. GalNAc-conjugated plasma clearance is driven by tissue distribution that occurs rapidly and extensively mainly to the liver and kidney. For this reason, GalNAc-conjugated ASOs have a faster plasma clearance than unconjugated ASOs due to a more efficient uptake by hepatocytes (over 5-fold) [19].

The three GalNAc sugars are cleaved off one after another by lysosomal *N*-acetyl-β-glucosaminidase, and then, the lysosomal DNase II (deoxyribonuclease II) remove the linker from the ASO. This hydrolysis creates an active phosphate species which is rapidly deactivated either by alkaline phosphatase or being esterified with one of the exposed hydroxyl groups to form cyclized metabolites. Upon further oxidation, these linker metabolites are eliminated as acids by biliary excretion. The phosphate-removed linkers are also metabolized by *O*- or *N*-dealkylation and eliminated by renal excretion [33].

Similar to unconjugated ASOs, renal excretion of GalNAc-conjugated ASOs is limited due to their high protein binding. Despite that, no protein binding-related DDIs would be expected for GalNAc-conjugated ASOs with small drug molecules. On the other hand, DDIs may occur when two or multiple ASOs are co-administered. Studies in animals have shown that an excipient ASO may enhance the pharmacological activity of another ASO, probably via protein binding displacement in the intracellular space [34,35]. However, the clinical relevance of these findings remains to be elucidated.

The major species excreted in urine is the unconjugated ASO, which represents only 1% of the total dose administered, together with various short nucleotide metabolites, as observed for unconjugated ASOs dosed alone [36]. After subcutaneous administration, 25.7% of the radioactive GalNAc is recovered in urine and 71.7% in the feces [37].

In vitro studies demonstrated that GalNAc-conjugated ASOs are not inhibitors nor inducers of major cytochrome P450 isoforms (e.g., CYP1A2, CYP2B6, and CYP3A4) and are not substrates or inhibitors of uptake or efflux membrane transporters (e.g., OATP, OAT, MDR1, etc.) [38,39]. Thus, ASOs and GalNAc-conjugated ASOs are predicted to have no DDIs with small drug molecules and to be safely administered in patients with chronic kidney disease (CKD) and ESRD without any significant drug accumulation [18,40].

The clearance of mAbs and ASOs does not involve CYP450 activity or interaction with cell membrane transporters, such as P-glycoprotein (P-gp), therefore their pharmacokinetic interactions with small molecule drugs can be excluded. The possible pharmacodynamic interaction with drugs affecting the coagulation cascade or the platelet activities remains to be established. Although the inhibitors of FXIa are considered safe in terms of risk of major bleeding [7,8,15], a clinically relevant interaction with antiplatelets, aspirin, or non-steroidal anti-inflammatory drugs (NSAIDs) cannot be excluded.

## 3. DOAC and Small Drug Molecules Anti FXIa: Potential Differences on DDIs

Differently from mAbs, small molecules can be cleared by means of CYP450 enzyme activities and by binding to drug transporters, such as P-gp, OATPs, OATs, and OCTs. Preclinical results indicate that milvexian is metabolized by CYP3A4/5, and it is a substrate of P-gp but not OATP [41]. The renal excretion ranged from 6.9% to 17.8% of the administered dose, and it is lower in the fasted state (6.9–12.3%) than in the fed state (16.3–17.8%). This difference is more likely due to the higher oral bioavailability in the presence of food [42]. The total exposure to milvexian, after a single oral 60 mg dose, was 41% and 54% greater in participants with eGFR values of 30 and 15 mL/min/1.73 m^2^, respectively, than in subjects with normal renal function. The median time to maximum concentration (T_max_) was similar for the three groups (4.5–5.0 h), and the half-life increased for participants with moderate (18.0 h) or severe (17.7 h) renal impairment compared with those with normal renal function (13.8 h) [43]. Thus, according to the low renal excretion, a single dose of milvexian 60 mg was safe and well tolerated in participants with moderate or severe renal impairment [43].

Asundexian was shown to be predominantly excreted via feces and, to a lower extent, in urine (80.3% and 20.3%, respectively), primarily either as a product of amide hydrolysis (~47%) or as an unchanged drug (~37%), with oxidative biotransformation playing a minor role (~13%) [44]. More recently, an in vitro analysis determined that asundexian is a P-gp substrate, and it is predominantly metabolized via carboxylesterase 1 and, to a lesser extent, via CYP3A4 [45]. Asundexian did not affect CYP1A2, CYP2A6, CYP2B6, CYP2C19, CYP2E1, CYP2J2, and CYP3A4 activity, while a weak inhibitory potential on CYP2C8, CYP2C9, CYP1A1, and CYP2D6 was observed [46]. Thus, both milvexian and asundexian are both P-gp substrates as the currently approved DOACs (dabigatran, edoxaban, apixaban, and rivaroxaban) (Table 2), and therefore, strong inhibitors of this drug transporter can increase absorption and exposure of these anticoagulants and the risk of bleeding [2]. On the other hand, an induction of P-gp can reduce their absorption and expose patients to thrombosis.

The most likely mechanism of pharmacokinetic interaction for all DOACs consists of significant gastrointestinal re-secretion over a P-gp transporter after absorption in the gut and in their renal clearance [48]. The intensity of the inhibition or induction of P-gp transporters can predict the entity of the change in drug exposure (Table 3).

Dabigatran and edoxaban seem to be less influenced by CYP450 inhibitors and inducers, since their metabolism does not involve this enzymatic pathway [50]. On the contrary, rivaroxaban and, to a lesser extent, apixaban are metabolized by CYP3A4, thus leading to a possible additional mechanism of DDI beyond the modulation of P-gp function (Table 2 and Table 3).

Individual variability of DOAC plasma concentrations may significantly impact the interaction with P-gp inhibitors or inducers. Thus, dabigatran, which shows a low oral bioavailability, and rivaroxaban, whose gastrointestinal absorption is strongly increased by the presence of food [2], may be more exposed to clinically significant DDIs.

The anti FXIa are also substrates for P-gp and, thus, can undergo similar DDIs with DOACs [2]. In addition, both milvexian and asundexian are metabolized by CYP3A4 and thus can interact with drugs that influence this metabolic pathway (inducers or inhibitors) (Table 2 and Table 3). The gastrointestinal absorption of both drugs is partially influenced by the presence of food, with a higher bioavailability for milvexian and lower for asundexian. However, these effects appear to have a lower impact compared to rivaroxaban, whose absorption is strongly reduced under fasting conditions.

Another level of differentiation between the DOAC and the new oral anti FXIa is the renal excretion. Milvexian and asundexian are marginally eliminated by the kidney and thus can be potentially administered in patients with moderate or severe renal impairment (Table 2), as documented by the results of a clinical trial [43].

## 4. Clinical Evidence of DDI with Milvexian and Asundexian

The potential effect of asundexian to inhibit and/or induce CYP3A4 over time was investigated in a nonrandomized, open-label study. The AUC and maximum plasma concentration (C_max_) of midazolam, a sensitive CYP3A4 substrate [51], and its metabolite, α-hydroxymidazolam, were determined before and after the coadministration of midazolam with a single or multiple doses of asundexian [52]. CYP3A4 metabolizes midazolam to α-hydroxymidazolam, thus its inhibition should determine a higher exposure of the administered drug and lower plasma levels of its metabolite.

The time to reach the C_max_ (T_max_) of midazolam and α-hydroxymidazolam was rapid (0.5–0.75 h) and was not affected by the co-administration of asundexian. Further, AUC, C_max_, and the half-life (T_1/2_) of midazolam and α-hydroxymidazolam were shown to be similar across the treatment groups. The AUC ratios of α-hydroxymidazolam to midazolam were comparable before and after the administration of asundexian at 25 mg and 75 mg (Table 4). Following repeated dosing with asundexian 25 mg once daily for 9 consecutive days, an increase in AUCs has been observed for midazolam and α-hydroxymidazolam by 6.3% and 6.0%, respectively. A slightly higher effect was observed with 75 mg of asundexian with an increase in the AUC for midazolam of 17% and a decrease in the AUC for α-hydroxymidazolam of 7.2%.

This trial demonstrated that asundexian 25 mg or 75 mg showed no indication of any clinically relevant inhibition and/or induction of CYP3A4 in healthy volunteers.

A second phase I clinical trial was performed to investigate the pharmacokinetics of asundexian upon co-administration with the two P-gp inhibitors, itraconazole and verapamil, with a strong and moderate inhibitory capacity towards CYP3A4, respectively, (Table 3) or with the moderate CYP3A4 inhibitor fluconazole, with no effect on P-gp.

Itraconazole increased the mean asundexian AUC by 103% (ratio: 2.0; 90% CI: 1.9–2.2) with a prolongation of the T_1/2_ from 16.2 to 28.9 h (Table 5). No effect on C_max_ of asundexian was observed in response to itraconazole administration. A less potent CYP3A4 inhibitor, verapamil, increased asundexian exposure (AUC) by 75.6% (ratio: 1.75, 90% CI: 1.66–1.85). Also, in this case, the effect on C_max_ was less pronounced (Table 5). Terminal T_1/2_ increased from 13.9 to 22.6 h compared with asundexian alone. Co-administration with fluconazole had a less pronounced effect on asundexian AUC and C_max_, with a 16.8% and 3.5% increase, respectively. Terminal half-life was comparable between treatments (15.5 h [with fluconazole] vs. 13.9 h [asundexian alone]).

The CYP3A4 and P-gp inducer carbamazepine reduced the concentrations of asundexian accelerating the elimination rate. Median T_max_ was slightly shorter, at 2.0 h with carbamazepine compared with 3.0 h for asundexian alone. The AUC of asundexian decreased by 44.4% (ratio: 0.56, 90% CI: 0.50–0.61) with concomitant administration of carbamazepine, whereas C_max_ was less affected (−18.2%) (Table 5).

This study observed a close correlation between the plasma concentration of asundexian and activated partial thromboplastin time (aPTT) or FXIa activity, indicating a direct inhibition of the coagulation cascade. APTT was measured via kaolin trigger, while FXIa was measured using a kaolin trigger and a proprietary fluorogenic readout [45]. Accordingly, the aPTT and FXIa activity returned to baseline levels after 48 h, while both parameters were still affected in the presence of itraconazole, suggesting a longer duration of action of asundexian [45]. Co-treatment with verapamil or fluconazole yielded intermediate results, according to their lower capacity to inhibit CYP3A4 and P-gp. On the contrary, the induction of CYP3A4 and P-gp by carbamazepine determined a fastest return to baseline for both pharmacodynamic markers (aPTT and FXIa) with values approaching baseline at 24 h post-dose [45].

Thus, the effect of itraconazole and verapamil on asundexian exposure may be related to a decrease in systemic CYP3A4-mediated biotransformation together with renal/non-renal P-gp driven excretion. In line with this hypothesis, asundexian renal clearance increased and decreased after the administration with P-gp and CYP3A4 inducers and inhibitors, respectively, but it was not affected by CYP3A4 inhibition alone as observed with fluconazole (Table 5).

Taken together, these results indicate a possible DDI of asundexian with drugs having a combined inhibitory effect on both P-gp and CYP3A4 (itraconazole and verapamil). However, only a minor effect was observed in response to moderate CYP3A inhibition (fluconazole). A lack of efficacy of asundexian can also be predicted in the presence of strong P-gp and CYP3A4 inducers, i.e., carbamazepine.

Similar DDI clinical studies have been performed with milvexian, where the effect of rifampicin (CYP3A4 and P-gp inducer) and two CYP450 inhibitors (itraconazole (strong) and diltiazem (moderate)) on the pharmacokinetic of milvexian were investigated (Table 6) [53,54].

These studies were carried out considering the preclinical data, demonstrating that milvexian is a substrate of CYP3A4/5 and P-gp [41]. Single dose of rifampicin does not affect the exposure of milvexian; however, following repeated doses the T_1/2_ was shorter (9 h vs. 13 h), as T_max_ values and a significant decrease in both C_max_, and AUC was observed with their values equal to 22% and 15%, respectively, compared to milvexian alone [54] (Table 6).

It has been established that acute administration of rifampin results in potent inhibition of hepatic OATP1B uptake transporters, and thus, the fact that a single dose of rifampicin did not affect milvexian exposure indicated that this drug is not an OATP1B substrate, as observed in vitro [41]. On the contrary, after multiple doses of rifampicin, a strong induction of CYP3A4 and P-gp can occur. Under this treatment, a significant reduction in C_max_ of milvexian was observed, indicative of a high level of induction of CYP3A and P-gp in the gut, leading to a limited systemic absorption of milvexian. Consistent with the lower plasma concentration of milvexian following repeated doses of rifampin, the activity of two pharmacodynamic biomarkers, aPTT or FXI, were minimally affected compared to when milvexian was administered alone [54]. 

The effects of strong and moderate CYP3A inhibitors, itraconazole and diltiazem, respectively, on the pharmacokinetic and pharmacodynamic properties of milvexian has also been assessed [53]. Considering the different inhibitory potency towards CYP3A4, the AUC of milvexian increased by 3.8-fold and by 38% with itraconazole and diltiazem, respectively. C_max_ was 28% higher in the presence of itraconazole (Table 6) and by 9.6% with diltiazem, compared with milvexian alone (Table 6). Finally, the T_1/2_ values were approximately 1.5- and 1.1-fold longer with itraconazole and diltiazem co-administration, respectively, while T_max_ values were similar in the two treatment arms [53]. As expected, aPTT was prolonged by milvexian administration either alone or when co-administered with itraconazole or diltiazem, with maximal mean aPTT prolongation occurring near C_max_. As observed with asundexian, the magnitude of aPTT prolongation was directly dependent by milvexian plasma concentration, and thus, a longer effect on aPTT was seen in the presence of itraconazole [53]. These results confirmed that milvexian is a substrate for CYP3A4 and P-gp. However, the impact of a strong CYP3A4 inhibitor was below 5-fold by the observed increases in milvexian exposure, and the impact of a moderate CYP3A4 inhibitor was less than 2.5-fold. This suggests that milvexian would not be considered a sensitive CYP3A4 substrate (i.e., AUC ≥ 5-fold) [55].

## 5. Conclusions

Considering the different pharmacokinetic characteristics of the three types of FXIa inhibitors, it appears clear that both mAbs and ASO clearance is not affected by kidney and liver impairment, and they do not undergo significant DDIs with conventional small molecule drugs. Thus, their use appears to be less problematic in older and multimorbidity patients; although, their very long half-life (one month and 2–4 weeks, respectively) may represent a critical issue in case of side effects, such as major bleeding. It remains, indeed, to be established that the pharmacodynamic interaction with drugs increase the risk of bleeding, such as antiplatelets, selective serotonin reuptake inhibitors (SSRI), NSAIDs, tyrosine kinase inhibitors, bevacizumab, and alemtuzumab [2].

On the contrary, the oral small drug molecule anti FXIa (milvexian and asundexian) may face similar DDI issues observed with the DOAC (dabigatran, apixaban, edoxaban, and rivaroxaban). For both drugs, we have results of dedicated clinical trials assessing their potential DDI with both inducers and inhibitors of CYP3A4 and P-gp. Although preclinical studies suggested a possible interaction dependent from the CYP3A4 pathway, milvexian and asundexian were demonstrated to undergo clinically relevant interactions with P-gp inhibitors and inducers, while their pharmacokinetics seem to be less influenced by modulators of CYP3A4. However, a potential advantage compared to the DOAC is represented by their very limited renal excretion, and thus, they may be administered in patients with CKD.

In conclusion, the anti FXIa could represent a valid alternative to DOAC for the treatment of patients with ACS, VTE, and stroke prevention of AF adding a new level of complexity in personalized medicine. The innovation in this field is not merely on the inhibition of a new coagulation factor, i.e., FXIa, but, most importantly, the development of biological therapies with different pharmacokinetic properties than small drug molecules. Nevertheless, the use and approval of these new therapies could be justified only after a clear demonstration of a better safety/efficacy profile than DOACs.

## Figures and Tables

**Table 1:** Molecular and pharmacological characteristics of anti FXIa inhibitors.

	mAbs (Abelacimab, Osocimab, and Xisomab)	Small Molecules (Asundexian and Milvexian)	ASOs (IONIS-FXIR_X_ and Fesomersen)
**Molecular weight**	150 KDa	<1 KDa	8–16 KDa
**Mechanism of action**	Bind FXIa or inhibit activation of FXI	Inhibit FXIa	Block mRNA of FXI translation
**Administration**	i.v. or s.c.	Oral	s.c.
**Administration frequency**	Monthly	Daily	Weekly/monthly
**Metabolism**	Proteolysis by reticuloendothelial system	CYP450	*N*-acetyl-glucosaminidase/DNase II
**Renal excretion**	No	Partially (6–18%)	Partially (25%)
**P-gp substrate**	No	Yes	No
**Type of DDI**	Pharmacodynamic	Pharmacodynamic/Pharmacokinetic	Pharmacodynamic

**Table 2:** Pharmacokinetic and pharmacodynamic characteristics of oral anti thrombin, FXa, and FXIa. Table has been modified from Ferri, N. et al., 2022 [2] with data on milvexian and asundexian derived from Perera, V. et al., 2022 [42] and from Roehrig, S. et al., 2023, Piel, I. et al., 2023, and Kanefendt, F. et al., 2023 [44,46,47].

Drug	Dabigatran	Rivaroxaban	Apixaban	Edoxaban	Milvexian	Asundexian
Target	Thrombin	FXa	FXa	FXa	FXIa	FXIa
Ki (nmol/L)	4.5	0.4	0.08	0.56	0.11	1.00
Bioavailability	6.5%	80%	50%	60%	ND	94%
Effect of food	Prolonged but not reduced	Increased (mainly with 20 mg)	None	None	Partially increased	Partially reduced
Vd (L)	60–70	50	21	>300	347	NA
Proteins bound	35%	>90%	87%	40–59%	92%	94%
Prodrug	Yes	No	No	No	No	No
T_max_ (h)	1–3	2–4	3–4	2	3–4	2.5–3
T_½_ (h)	12–17	5–9	8–15	8–11	9–10	14–17
Metabolism (CYP)	Conjugation	3A4 (18%), 2J2, Independent from CYP	CYP3A4 (25%), CYP1A2, CYP2J2, CYP2C8, CYP2C9, CYP2C19	3A4 (<4%)	3A4	3A4 Modest 2C8, 2C9, 1A1 and 2D6 inhibitor Modest CYP3A4 inducer
Substrate P-gp	Yes (only prodrug)	Yes	Yes	Yes	Yes	Yes
Substrate of other transporters	NA	BCRP/ABCG2	BCRP/ABCG2	NA	No OATP	NA
Renal excretion	80%	65%	27%	35%	7–18%	6%
Posology	BID	OD	BID	OD	BID/OD	BID/OD

BID: bis in die; OD: once daily; BCRP: breast cancer resistance protein; ABCG: ATP Binding Cassette Subfamily G Member; OATP: Organic Anion Transporting Polypeptides; NA: not available.

**Table 3:** Inducers and inhibitors of CYP3A and P-gp. Modified from Corsini et al. [49].

	P-gp Inhibitor	Non-P-gp Inhibitor	P-gp Inducer
**Strong CYP3A inhibitor**	itraconazole, ketoconazole, clarithromycin, lopinavir, indinavir, ritonavir, telaprevir	voriconazole, fluconazole	
**Moderate CYP3A inhibitor**	erythromycin, verapamil, diltiazem, dronedarone	not identified	doxorubicin
**Weak CYP3A inhibitor**	lapatinib, quinidine, cyclosporine, felodipine, azithromycin, ranolazine, ticagrelor, chloroquine, hydroxychloroquine	cimetidine	vinblastine
**CYP3A Inducers**			carbamazepine, phenytoin, phenobarbital, rifampin, dexamethasone, tocilizumab, St. John’s Wort

**Table 4:** AUC ratio of midazolam and α-hydroxymidazolam following treatment with asundexian.

Treatment	Analyte	AUC Ratio Day 1/Day 1 (90% CI)	AUC Ratio Day 10/Day 1 (90% CI)
Asundexian 25 mg OD + midazolam	Midazolam	1.04 (0.97–1.12)	1.06 (0.99–1.14)
	α-Hydroxymidazolam	1.07 (0.97–1.17)	1.06 (0.96–1.16)
Asundexian 75 mg OD + midazolam	Midazolam	1.04 (0.98–1.12)	1.17 (1.10–1.26) *
	α-Hydroxymidazolam	0.99 (0.92–1.06)	0.99 (0.92–1.06)

Modified from Kubitza et al., 2022 [52]. * Ratio above 1.25.

**Table 5:** Pharmacokinetic parameters of asundexian in the presence or absence of itraconazole, verapamil, fluconazole, or carbamazepine.

Parameter	Asundexian	Asundexian + Itraconazole	Ratio Asundexian +Itraconazole/Asundexian		
AUC (μg h L^−1^)	6920	14,000	2.02		
C_max_ (μg L^−1^)	377	387	1.03		
T_max_	3.48	2.49	0.71		
T_1/2_	16.2	28.9	1.78		
CL/F	3.61	1.78	0.49		
CL_R_	0.307	0.180	0.59		
**Parameter**	**Asundexian**	**Asundexian + verapamil**	**Ratio asundexian + verapamil/asundexian**	**Asundexian + fluconazole**	**Ratio asundexian + fluconazole/asundexian**
AUC (μg h L^−1^)	6360	11,200	1.76	7430	1.17
Cmax (μg L^−1^)	347	396	1.14	359	1.03
T_max_ (h)	3.47	3.00	0.87	3.45	0.99
T_1/2_ (h)	21.8	22.6	1.04	15.5	0.71
CL/F (L h^−1^)	3.93	2.24	0.57	3.37	0.86
CL_R_ (L h^−1^)	0.297	0.215	0.72	0.313	1.05
**Parameter**	**Asundexian**	**Asundexian + carbamazepine**	**Ratio asundexian + carbamazepine/asundexian**		
AUC (μg h L^−1^)	11,500	6380	0.55		
Cmax (μg L^−1^)	623	509	0.82		
T_max_ (h)	2.00	3.00	1.50		
T_1/2_ (h)	14.4	11.0	0.76		
CL/F (L h^−1^)	4.36	7.84	1.80		
CL_R_ (L h^−1^)	0.265	0.377	1.42		

Asundexian was administered as 25 mg oral single dose alone or in combination with itraconazole and verapamil, while the study with carbamazepine used 50 mg oral dose. Modified from Kanefendt F. et al., 2023 [45]. CL/F, apparent plasma clearance of drug after extravascular administration; CL_R_, renal clearance of drug.

**Table 6:** Pharmacokinetic parameters of milvexian in the presence or absence of rifampicin, itraconazole, or diltiazem.

Parameter	Milvexian	Milvexian Following Repeated Doses of Rifampicin	Ratio Milvexian + Rifampicin/Milvexian
C_max_ (ng/mL)	599	132	0.22
AUC (ng·h/mL)	6153	923	0.15
T_max_ (h)	3.5	4.0	1.14
T_1/2_ (h)	13.21	8.85	0.67
**Parameter**	**Milvexian**	**Milvexian + itraconazole**	**Ratio milvexian + itraconazole/milvexian**
C_max_ (ng/mL)	229	293	1.28
AUC (ng·h/mL)	2144	5342	2.49
T_max_ (h)	3.0	4.0	1.33
T_1/2_ (h)	11.6	17.1	1.47
**Parameter**	**Milvexian**	**Milvexian + diltiazem**	**Ratio milvexian + diltiazem/milvexian**
C_max_ (ng/mL)	248	272	1.10
AUC (ng·h/mL)	2220	3059	1.38
T_max_ (h)	3.0	4.0	1.33
T_1/2_ (h)	12.3	13.6	1.11

Modified from Perera V. et al., 2022 and Perera V. et al., 2022 [53,54].
